# Antioxidant Potential of Polyphenolic Compounds, Sources, Extraction, Purification and Characterization Techniques: A Focused Review

**DOI:** 10.1002/fsn3.71259

**Published:** 2025-12-09

**Authors:** Zubair Ahmad, Abdur Rauf, Ilkay Erdogan Orhan, Mohammad S. Mubarak, Zuneera Akram, Md. Rezaul Islam, Muhammad Imran, Zehra Edis, Benod Kumar Kondapavuluri, Lakshmi Thangavelu, Muthu Thiruvengadam

**Affiliations:** ^1^ Department of Chemistry University of Swabi Ambar Khyber Pakhtunkhwa Pakistan; ^2^ Department of Pharmacognosy, Faculty of Pharmacy Lokman Hekim University Ankara Türkiye; ^3^ Department of Chemistry The University of Jordan Amman Jordan; ^4^ Department of Pharmacology, Faculty of Pharmaceutical Sciences Baqai Medical University Karachi Pakistan; ^5^ Department of Pharmacy, Faculty of Allied Health Sciences Daffodil International University Dhaka Bangladesh; ^6^ Chemistry Department, Faculty of Science King Khalid University Abha Saudi Arabia; ^7^ Center of Medical and Bio‐Allied Health Sciences Research Ajman University Ajman UAE; ^8^ Dr. D.Y. Patil Medical College, Hospital and Research Centre Pune India; ^9^ Center for Global Health Research Saveetha Medical College and Hospital, Saveetha Institute of Medical and Technical Sciences Chennai India; ^10^ Department of Crop Science College of Sanghuh Life Science, Konkuk University Seoul Republic of Korea

**Keywords:** antioxidants, extraction techniques, natural products, polyphenolic compounds

## Abstract

Polyphenolic compounds, which are abundant phenolic metabolites in plants, have attracted significant attention because of their diverse biological activities and robust antioxidant properties. This review uniquely describes the extraction characterization, and application pipeline for polyphenols, emphasizing their mechanistic antioxidant actions and translational challenges. Between 1979 and 2025, approximately 141 relevant studies were critically examined, covering the sources, extraction and purification methods, structural diversity, and biological implications of polyphenols. Special attention is given to bioavailability, dose‐dependent effects, and subclass‐specific activities. Various extraction techniques, including solvent extraction, supercritical fluid extraction, and solid‐phase extraction, have been evaluated along with advanced characterization approaches such as spectroscopy and chromatography. The antioxidant mechanisms of polyphenols, ranging from radical scavenging and metal chelation to modulation of signaling pathways, are described, along with their broad applications in nutrition, medicine, and industry. This focused synthesis highlights not only the current state of knowledge, but also identifies key research gaps in clinical validation, formulation strategies, and industrial adoption, providing a forward‐looking perspective for future polyphenol research.

## Graphical abstract

This review highlights the antioxidant potential of polyphenolic compounds derived from diverse plant sources. It outlines extraction and purification methods, advanced spectroscopic and chromatographic characterization techniques, and underlying antioxidant mechanisms such as radical scavenging, metal chelation, and signalling modulation. The study emphasizes bioavailability challenges, mechanistic insights, and future prospects for polyphenol‐based nutraceuticals, pharmaceuticals, and functional foods.

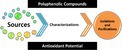

## 1 Introduction

There has been a growing interest in natural bioactive compounds, especially those known for their antioxidant properties (Bernhoft 2010; Purewal et al. 2023). Antioxidants are essential for neutralizing harmful free radicals in the body, and are associated with various diseases and aging processes. Numerous natural sources such as fruits, vegetables, herbs, and spices contain significant amounts of antioxidants, making them increasingly popular in scientific research and consumer products. These compounds have potential health benefits, and offer opportunities for the development of new pharmaceuticals, supplements, and functional foods. Among these, polyphenolic compounds have emerged as focal points of scientific exploration because of their potential health‐promoting effects (Sun and Shahrajabian 2023).

Polyphenols, which are abundant secondary metabolites in plants, play a crucial role in mitigating oxidative stress and promoting overall health (Rudrapal et al. 2022, 2024). These compounds exhibit multifaceted properties, ranging from free radical scavenging to modulation of intracellular signaling pathways. The antioxidant properties of polyphenols, primarily through their role in neutralizing harmful compounds linked to various diseases, are achieved by the donation of electrons or hydrogen atoms to reactive oxygen and nitrogen species (Lv et al. 2021). Polyphenols' potent redox properties neutralize the species involved in the onset and progression of multiple diseases. Consequently, the potential of polyphenolic compounds to counteract oxidative damage has ignited investigations into their utility as natural remedies to combat oxidative stress‐related disorders (Vuolo et al. 2019). As the scientific understanding of polyphenols continues to evolve, researchers are exploring various aspects such as their bioavailability, metabolism, and specific mechanisms of action in different diseases. Accordingly, this comprehensive review delves into the polyphenolic compounds isolated from plants, focusing on their antioxidant potential. This study investigated the complicated mechanisms by which polyphenols exert their antioxidant effects, revealing the molecular intricacies that underpin their therapeutic potential. Although the antioxidant capacity of polyphenolic compounds has been widely acknowledged, assessing their efficacy remains a complex task. State‐of‐the‐art methodologies have been developed to gauge the antioxidant properties of these compounds. However, inherent limitations persist.

In the subsequent sections of this review, we aimed to dissect the various facets of polyphenolic compounds as antioxidants. This review provides a comprehensive understanding of their roles in modern science and industry by exploring their diverse classifications, sources, mechanisms, measurement techniques, and potential applications (Zaini et al. 2022). As this review navigates multifaceted landscapes, we are confronted with several questions. How do different polyphenolic compounds exhibit varying degrees of antioxidant activities? What are the specific mechanisms by which these compounds interact with cellular components to mitigate oxidative stress? How can their potential to ameliorate the burden of oxidative stress‐related diseases be harnessed? In addressing these questions, it is evident that the health benefits attributed to polyphenolic antioxidants extend beyond their intrinsic redox properties (Fukumoto and Mazza 2000). These compounds can modulate gene expression, influence enzymatic activities, and regulate inflammatory responses (Yahfoufi et al. 2018). Interpreting the intricate interactions underlying these effects could potentially unlock novel therapeutic avenues for various ailments. However, harnessing the full potential of polyphenolic compounds is a challenge.

Furthermore, the wide variability in polyphenol content across different plant sources necessitates a nuanced approach to understand their diverse effects (D'Archivio et al. 2007). The backdrop of these challenges lies in the numerous opportunities for innovation. Within this context, the integration of advanced analytical techniques, including metabolomics and systems biology, holds promise for unraveling the intricate mechanisms of polyphenolic action. Precise approaches that consider individual genetic makeup, lifestyle factors, and disease contexts could pave the way for personalized polyphenol‐based interventions. As our knowledge expands, it is evident that the landscape of polyphenolic antioxidants is dynamically shaped by ongoing research (Tresserra‐Rimbau et al. 2018). Furthermore, the methods employed to measure this potential and critically assess their strengths and limitations have also been addressed. Based on established and emerging research, this review examines the progress in understanding, harnessing, and applying polyphenolic antioxidants.

## 2 Polyphenolic Compounds

Polyphenolic compounds form diverse and intricate bioactive molecules that are characterized by their distinct chemical structures and functions (Tazzini 2014). Within this, the world of polyphenols is rich and diverse, encompassing various subclasses with unique chemical structures and biological activities (Zeng et al. 2023). The vast diversity of polyphenolic compounds found in nature is particularly notable. Over 8000 polyphenolic compounds have been identified across various plant species (Gębalski et al. 2023) and researchers have only scratched the surface of this complex chemical landscape. Phenolic compounds are diverse plant secondary metabolites that have a wide range of structures. These structures can vary from relatively basic phenolic acids to polyphenols, such as flavonoids, which consist of multiple groups, and even polymeric compounds derived from these classes. Phenolic compounds play a crucial role in determining the quality of plant‐based food. They are responsible for the color of red fruits, juices, and wines, as well as enzyme browning substrates, and contribute to taste characteristics. Specifically, astringency is attributed to the formation of salivary protein aggregates by polyphenols, a process that may be implicated in protecting against their detrimental effects on nutrition. Phenolic chemicals are believed to contribute to health advantages associated with the consumption of fruits and vegetables. Plant phenolics are converted to a diverse range of chemicals during food processing and storage. Although techniques for analyzing phenolic compounds with low molecular weights are well established, analyzing polymeric compounds remains a challenge. Their significant interactions with plant cell wall components limit the extraction of polymeric phenolics. Furthermore, their characteristic of being polydispersity leads to inadequate resolution and detection, particularly for derived structures such as oxidation products. Nevertheless, recent advancements in analytical methods have enabled improvements in the structural characterization of these elements (Cheynier 2012).

### 2.1 Biosynthesis of Polyphenolic Compounds

The biosynthesis of plant phenolic compounds begins with the amino acid phenylalanine, which is derived from the shikimate pathway (Kaur et al. 2023; Tak et al. 2023). Once synthesized, phenolic compounds undergo various modifications to form diverse structures and chemical properties. One common modification is the conjugation of phenolic compounds with sugar residues, which results in the formation of glycosides. This conjugation enhances the solubility and stability of phenolic compounds (Tariq and Ahmed 2023) and influences their bioavailability and biological activity (Hamid et al. 2023). Additionally, direct linkage of sugars to aromatic carbons can occur to form glycosidic bonds. Moreover, phenolic compounds in plants can form complex structures through intermolecular linkages, creating dimers, oligomers, and polymers, thereby enhancing the chemical diversity of these compounds (Hamid et al. 2023; Shahidi and Dissanayaka 2023).

### 2.2 Classification of Polyphenolic Compounds

**FIGURE 1:**
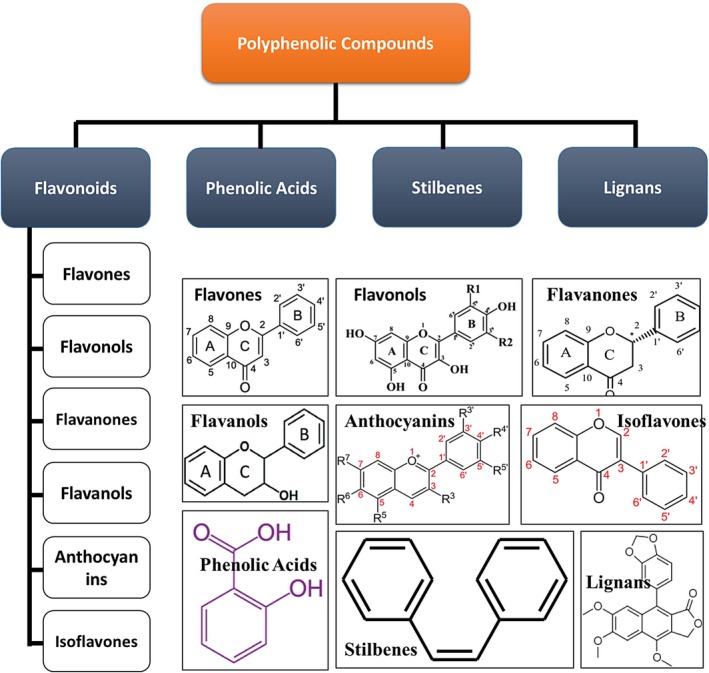
Classification and chemical structure of polyphenolic compounds.

Polyphenols can be categorized based on the number of phenol rings and the structural elements that bind these rings. The main classes of compounds are phenolic acids, flavonoids, stilbenes, and lignans. Their chemical structures are illustrated in Figure 1. In this section, notable types of polyphenolic compounds are discussed that unravel their structural diversity and shed light on their diverse roles as antioxidants.

#### 2.2.1 Flavonoids

Flavonoids represent a vanguard of polyphenolic diversity and are the most abundant and extensively studied subclasses of these compounds. Phenolic compounds found in various plants are naturally occurring compounds with diverse compositions in fruits, vegetables, cereals, bark, roots, stems, flowers, tea, and wines. Flavonoids are naturally occurring substances that are widely recognized for their health benefits, and ongoing efforts are being made to separate their components. Flavonoids are increasingly being recognized as essential constituents of several nutraceuticals for pharmacological, therapeutic, and cosmetic applications. These effects are attributed to their anti‐oxidative, anti‐inflammatory, anti‐mutagenic, anti‐carcinogenic, and antidiabetic potential, as well as their ability to regulate crucial cellular enzyme activity (Hussain et al. 2021; Panche et al. 2016). Polyphenolic phytochemicals produced by plant secondary metabolism are low‐molecular‐weight compounds with significant biological effects. They possess a wide range of characteristics that are advantageous to human health by interacting with several cellular targets relevant to important cell signaling pathways in the body (Singh et al. 2014). The intricate structures of these compounds, consisting of two benzene rings connected by a three‐carbon bridge, exhibit a wide range of chemical diversities and biological activities (Panche et al. 2016). Within the realm of flavonoids, six distinct subclasses emerge, each with its unique structural features and biological activities (Chen et al. 2023).

##### 2.2.1.1 Flavones

Flavones are compounds with a double bond in the C ring that contains apigenin and luteolin. These compounds have demonstrated antioxidant potential through various mechanisms, including free radical scavenging and chelation of metal ions. They are renowned for their beneficial antioxidant properties, and are distinctive in their ubiquitous presence in most fruits and vegetables. As most metabolic disorders are believed to arise from oxidative stress, recent research has demonstrated the beneficial impact of flavones on oxidative stress‐related medical conditions. The diverse array of biological actions of flavones has piqued the interest of medicinal chemists, leading to the identification of many lead compounds in various disease domains (Verma and Pratap 2010). Flavones are naturally occurring compounds found in fruits and vegetables, and are often consumed unintentionally in our daily diet. These components have a beneficial influence on health without significant adverse consequences. The investigation of different techniques for the synthesis and structural modification of flavone rings has become a crucial objective for multiple research groups to study the various functions of flavones. Natural flavone monomers with diverse biological properties can be used to produce partially or fully synthetic flavone derivatives with distinct functional groups at different locations in the flavone skeleton (Singh et al. 2014).

##### 2.2.1.2 Flavonols

Flavonols, particularly quercetin, are widespread in plants and found in substantial quantities in fruits and vegetables. Furthermore, apart from their antioxidant properties, flavonols disrupt several biochemical signaling pathways, thereby affecting both physiological and pathological processes (Perez‐Vizcaino and Duarte 2010). The presence of a 3‐hydroxyflavone backbone characterizes flavonols and typically features hydroxyl groups at the 3‐position and/or 5‐position. Hydroxyl groups in positions 3, 5, and 7 in the C ring contribute to their potent scavenging of free radicals and ROS (Wang et al. 2006). The presence of these hydroxyl groups enhances antioxidant properties (Amić and Mastiľák Cagardová 2023; Chen et al. 2023). Some examples include quercetin, kaempferol, and myricetin. Flavonols, found in fruits, vegetables, tea, and red wine, are known for their potent antioxidant and anti‐inflammatory properties.

##### 2.2.1.3 Flavanones

Flavanones, distinctive polyphenols found in Citrus species, are primarily responsible for their beneficial effects. Plant species contain primary flavanones such as hesperetin, naringenin, eriodictyol, isosakuranetin, and their corresponding glycosides. Hesperetin and its derivatives are flavanones found in sweet orange, tangelo, lemon, and lime, whereas naringenin and its derivatives are exclusive to grapefruit and sour oranges. Advanced analytical methods, such as UPLC and mass spectrometry, have enabled the rapid and efficient estimation of flavanone levels in plant species and humans after ingestion (Khan and Dangles 2014). Flavanones, including naringenin and hesperidin, possess a 2,3‐dihydroflavone backbone and antioxidant properties that modulate enzymatic and signaling pathways involved in oxidative stress (Di Majo et al. 2005).

##### 2.2.1.4 Flavanols

Flavanols, also known as catechins, have 2,3‐dihydroflavan‐3‐ol backbones with hydroxyl groups at the 3‐ and 5‐positions, respectively. They are ubiquitous among all plant species. Their production often relies on light, resulting in their primary concentration in the tissues of the outer layer. Excluding onions, the flavonol content in freestanding leaves surpasses that in other parts of the same plant (Herrmann 1976). Flavanols, including catechins, are abundant in tea, cocoa, and berries, along with epicatechin, epigallocatechin, and their derivatives, which are potent antioxidants. Antioxidant properties are attributed to redox properties and interactions with cellular components (Hackman et al. 2008). They have also been recognized for their cardiovascular‐protective effects and potential anti‐cancer activities. Flavonols, such as fisetin, kaempferol, morin, myricetin, and quercetin, have been found to possess distinct antioxidant and anti‐inflammatory properties. The properties of the compounds are attributed to their crystal structure, with the presence of a 3′,4′‐catechol moiety in the B ring, indicating significant activity. The 4′‐OH group of the B ring significantly reduced the superoxide produced by xanthine/xanthine oxidase. However, the presence of an additional OH group at the ortho sites weakened the observed impact. This study found that F, Q, and MO were more effective as ROS inhibitors than MY and K because of their unique structural features in the B ring. The structural requirements for enhancing the free radical scavenging activity differ depending on the specific free radical. Flavonols effectively inhibited the production of nitric oxide (NO) in RAW264.7 murine macrophages without any noticeable toxicity, indicating their potential as antimicrobial agents. F, K, and Q exhibited dose‐dependent inhibition of iNOS mRNA expression and prostaglandin E2 synthesis, partially by reducing the NF‐κB signaling pathway activity. These findings indicate that flavonols, although having comparable structures, exhibit distinct antioxidant and anti‐inflammatory properties (Wang et al. 2006).

##### 2.2.1.5 Anthocyanins

Anthocyanins, derived from the Greek word anthos, meaning flower and kyanos (blue), are primary plant pigments with visual properties to the human eye. Their classification is based on a broad category of phenolic compounds known as flavonoids. These compounds are glycosides made from the polyhydroxy and polymethoxy derivatives of salts containing 2‐phenylbenzopyrylium or flavylium. Anthocyanins are endogenous chemicals responsible for the pigmentation of fruits, vegetables, and plants, ranking second only to chlorophyll and being the most significant category of visible plant pigments. In addition to providing color to plants, anthocyanins offer a range of health‐enhancing advantages by safeguarding plants against different oxidants through a multitude of processes. Nevertheless, anthocyanins have garnered less attention than other flavonoids (Kong et al. 2003). Anthocyanins are water‐soluble pigments that impart red, purple, and blue colors to fruits, flowers, and vegetables, respectively. Examples include cyanidin, delphinidin, and pelargonidin. These compounds have notable antioxidant potential, and are responsible for the vibrant hues of various fruits and vegetables. Anthocyanins contribute to the broad‐spectrum antioxidant activities of plant‐derived compounds (Kong et al. 2003; Wallace and Giusti 2015) and anti‐inflammatory properties and may contribute to cardiovascular health and cognitive function. Seventeen naturally occurring anthocyanidins or aglycones are influenced by hydroxyl groups, sugar types, location, and aliphatic/aromatic acids, resulting in variations among distinct anthocyanins (Kong et al. 2003). The higher plants used six anthocyanidins: Pg, Pn, Cy, Mv, Pt, and Dp. The glycosides Cy, Dp, and Pg are abundant in leaves, fruits, and flowers. The six predominant anthocyanidins found in plant consumable portions are cyanidin (50%), pelargonidin (12%), peonidin (12%), delphinidin (12%), petunidin (7%), and malvidin (7%). Anthocyanidin glycosides, including 3‐monosides, 3‐biosides, 3,5‐diglycosides, and 3,7‐diglycosides, are widely distributed and 2.5 times more common than 3,5‐diglycosides (Ren et al. 2001).

##### 2.2.1.6 Isoflavones

Isoflavones are structurally similar to flavonols but feature a 3‐phenylchromen‐4‐one backbone. It exhibits structural similarity to estrogens and is often referred to as a phytoestrogen. Genistein and daidzein are the prominent isoflavones. Isoflavones are primarily found in soybeans and soy products, and they are known for their estrogenic and antioxidant activities (Křížová et al. 2019; Ruiz‐Larrea et al. 1997). This investigation is currently underway to explore the potential role of isoflavones, a specific type of soybean protein, in cancer prevention and treatment. These compounds exhibit many biological and pharmacological effects, including estrogenic and anti‐estrogenic properties, participation in cell signaling, and modulation of cell growth and death. They have been linked to reduced instances of breast and prostate cancer, cardiovascular disorders, and osteoporosis. These effects are attributed to several pathways, including estrogenic characteristics, inhibition of protein tyrosine kinase, control of gene transcription, modification of transcription factors, antioxidants, and alteration of enzyme activity (Ren et al. 2001).

### 2.3 Phenolic Acids

Phenolic acids are a varied group of compounds, defined by the presence of carboxylic acid functional groups. Two important subclasses are hydroxycinnamic and hydroxybenzoic acids, both of which contribute significantly to the antioxidant properties of phenolic acids (Soares 2002; Tessema et al. 2023). Because of their strong antioxidant activity and additional health benefits, plant‐derived phenolics are considered an essential component of the human diet. Epidemiological studies have indicated that diets rich in antioxidant‐rich fruits and vegetables can lower the risk of chronic conditions such as cancer, diabetes, and cardiovascular disease. The antioxidant potential of these compounds largely depends on the number and position of the hydroxyl groups, with polyphenols recognized as the main dietary source of antioxidants. Peptenic acids, a subgroup of plant phenolics, show antioxidant effects owing to their resonance‐stabilized structures, which allow them to donate hydrogen atoms and neutralize free radicals. Overall, the antioxidant function of phenolic acids is linked to several mechanisms, including electron donation for radical quenching and singlet oxygen neutralization. In addition to their antioxidant role, phenolic acids have also been investigated for a wide range of health‐promoting effects, such as antibacterial, anticancer, anti‐inflammatory, and anti‐mutagenic properties (Kumar and Goel 2019).

#### 2.3.1 Hydroxycinnamic Acids

Ferulic and caffeic acids are examples of hydroxycinnamic acids that are widespread in plant foods (fruits, vegetables, grains, and coffee). Hydroxycinnamic acids are antioxidants that can neutralize free radicals and prevent oxidative reactions by donating hydrogen atoms to free radicals and binding transition metal ions, such as iron and copper, in the form of chelates. In addition, they regulate the cellular signaling pathways of oxidative stress and inflammatory responses, which are responsible for their antioxidant activities (Ou and Kwok 2004). Hydroxycinnamic acids (HCAs) are a family of phenolic acids that are mostly present in plants and are characterized by the basic chemical structure of the phenylpropanoid C_6_‐C_3_ backbone. These chemicals occur in most foods, including tea leaves, coffee, red wine, fruits, vegetables, whole grains (Teixeira et al. 2013). P‐coumaric, caffeic, ferulic, and sinapic acids are hydroxycinnamic acids with specific ecological importance. The massive diversity and plurality of these compounds render them essential for the biosynthesis of complex phenolic compounds. Hydrocarbon ammonias (HCAs) are secondary metabolites present in conjugated forms (amides, esters, and glycosides), mainly esters of hydroxy acids. Higher plants contain cinnamate esters; however, cinnamic acid amides are less widespread (Teixeira et al. 2013).

#### 2.3.2 Hydroxybenzoic Acids

Hydroxybenzoic acids, such as gallic acid and ellagic acid, occur in a range of plant foods, such as berries, nuts, and herbs. Similar to hydroxycinnamic acids, hydroxybenzoic acids exhibit antioxidant activity through multiple mechanisms. They can scavenge free radicals directly by producing hydrogen atoms or electrons, thus preventing oxidative damage to biomolecules. Moreover, hydroxybenzoic acids have metal‐chelating activities, which alleviate metal‐induced oxidative stress. In addition, these compounds have been implicated in the control of cellular signaling pathways in the oxidative stress response and inflammation, and thus have protective effects against oxidative damage (Sharma et al. 2018). Hydroxycinnamic and hydroxybenzoic acids are essential phenolic acids that have been identified as contributing to plant‐based foods because of their antioxidative effects, health‐promoting effects, and alleviation of the negative impact of oxidative stress‐associated illnesses. The addition of these compounds to the diet via the intake of fruits, vegetables, and other vegetable sources can contribute to general well‐being and prevent diseases (Pandey and Rizvi 2009).

### 2.4 Stilbenes

Stilbene is a plentiful structural framework found in nature and several compounds derived from stilbene have been extensively documented for their biological properties. The cardioprotective, potent antioxidant, anti‐inflammatory, and anticancer properties of (E)‐resveratrol and its stilbene derivatives have been extensively studied. Stilbene backbones have been widely researched for their effective chemotherapeutic capabilities against a variety of cancers. Science has been working on transforming stilbene structures to enable the development of new analogs with better anticancer activity and bioavailability. Within the last decade, the majority of stilbene derivatives produced in recent years have demonstrated significant anticancer activity in a range of cancer cell lines, and it was determined that the anticancer effects depend on the nature and position of the substituents on the stilbene backbone (De Filippis et al. 2017). Stilbenes are defined by a central 1, 2‐diphenylethylene core structure that is absent in other groups of polyphenolic compounds. Resveratrol is one of the most studied stilbenes and has been widely used owing to its promising health value. This substance has been associated with numerous cellular roles, such as activation of sirtuins and regulation of redox‐sensitive signaling pathways (Jeandet et al. 2023; Miura et al. 2000; Shen et al. 2009).

### 2.5 Lignans

Lignans are a fascinating group of compounds that possess diverse biological activities and health benefits. Lignans derived from phenylpropanoid precursors are abundant in various seeds, grains, and vegetables. They are characterized by their complex structures, which often feature multiple aromatic rings and linkages. Secoisolariciresinol, matairesinol, and enterolignans are notable examples in this group. Lignans are being studied for their potential antioxidant properties, which could help combat oxidative stress and reduce the risk of chronic diseases such as cardiovascular disease and certain types of cancer. Enterolignans and lignans have been studied for their roles in hormone regulation, particularly as phytoestrogens. Phytoestrogens are plant‐derived compounds that mimic or regulate the effects of estrogen, potentially enhancing hormone balance and cellular homeostasis (MacRae and Towers 1984; Saleem et al. 2005). Table 1 summarizes the structural diversity of polyphenolic compounds and their wide‐ranging antioxidant activities, bioavailability differences, and health implications, which are further explored in the following section on their antioxidant properties.

**TABLE 1:** Major classes of polyphenolic compounds, examples, sources, and functional notes.

Class	Examples	Key sources	Antioxidant mechanism/activity	Bioavailability notes
Flavonoids	Quercetin, catechins, hesperidin, luteolin	Fruits, vegetables, tea, wine	Radical scavenging, metal chelation, enzyme modulation, signaling pathway regulation	Moderate but variable; glycosides often improve solubility
Phenolic acids	Gallic acid, caffeic acid, ferulic acid	Berries, coffee, whole grains	Hydrogen donation, electron transfer, metal chelation	Generally higher absorption than flavonoids; rapid metabolism
Stilbenes	Resveratrol, pterostilbene	Grapes, peanuts, red wine	Sirtuin activation, anti‐inflammatory, ROS scavenging	Low systemic bioavailability due to rapid conjugation
Lignans	Secoisolariciresinol, matairesinol, enterolactone	Flaxseed, sesame, whole grains, legumes	Phytoestrogenic activity, antioxidant, hormone modulation	Converted by gut microbiota into enterolignans; bioavailability depends on microbiome
Anthocyanins	Cyanidin, delphinidin, malvidin	Berries, cherries, red cabbage, purple corn	Pigment antioxidants, free radical scavenging, signaling modulation	Poor stability, highly affected by pH, heat, and processing

## 3 Antioxidant Properties of Polyphenolic Compounds

Polyphenols are secondary metabolites found in fruits, vegetables, whole grains, nuts, and beverages like tea and wine (Handique et al. 2002). The term “polyphenols” encompasses various compounds, including flavonoids, phenolic acids, lignans, stilbenes, and anthocyanins, each with a unique chemical structure and biological activity (Tazzini 2014). These compounds are recognized for their ability to counteract ROS and RNS, collectively known as free radicals, thereby mitigating oxidative stress‐induced damage in biological systems (Lv et al. 2021). Polyphenols exhibit strong antioxidant properties, effectively countering the reactivity of free radicals via electron or hydrogen atom donations. The antioxidant activity of flavonoids is attributed to their structural features, including highly conjugated systems and specific hydroxylation patterns, which significantly enhance their potent antioxidant properties. Through these mechanisms, polyphenols effectively curtail the generation of free radicals, thereby diminishing the rate of oxidative processes (Gebicki and Nauser 2021). This is achieved by thwarting the formation of or neutralizing the active species and precursors responsible for initiating free‐radical cascades. Notably, polyphenols often play a crucial role as direct scavengers of radicals in lipid peroxidation chain reactions, assuming the role of “chain breakers”. In this role, they extend electrons to free radicals, nullifying their reactivity. Consequently, polyphenols become stable, have fewer reactive radicals, effectively interrupt chain reactions, and ultimately confer stability to the oxidative process (Kurutas 2015). Flavonoids, flavones, phenolic acids, and other polyphenolic compounds are known for their exceptional antioxidant potential, which is pivotal for safeguarding various aspects of human health. These compounds, originating from various natural sources, have numerous beneficial effects by reducing oxidative stress and preventing the harmful effects of reactive oxygen species (ROS) and free radicals (Swallah et al. 2020). Understanding the intricate mechanisms underlying their antioxidant properties is essential to understand their profound physiological implications. Flavonoids, a prominent subclass of polyphenolic compounds, exhibit strong antioxidant activity owing to their complex molecular structure. Direct radical scavenging is a fundamental mechanism, wherein the hydroxyl groups within their structures readily interact with and stabilize ROS and free radicals through hydrogen atoms or electron donation (Bhattacharya 2015). Flavonoids can inhibit the generation of ROS through Fenton and Haber‐Weiss reactions by chelating transition metal ions such as iron and copper. These compounds stimulate the production of endogenous antioxidant enzymes such as SOD and catalase, thereby enhancing cellular defense against oxidative stress (Cotelle 2001). Flavonoids also exhibit anti‐inflammatory properties, indirectly enhancing their antioxidant potential by suppressing ROS production during the inflammatory response (Elisha et al. 2016). Flavones are a noteworthy subclass of flavonoids that are known for their potent antioxidant properties. These compounds are particularly effective in preserving mitochondrial integrity and minimizing ROS production within the cellular powerhouses. Furthermore, flavones modulate various signaling pathways, including the Nrf2‐Keap1 pathway, which governs antioxidant enzyme transcription (Moosavi et al. 2015). By activating Nrf2, flavones bolster the cellular antioxidant defense system, contributing to their overall protective effects (Qi et al. 2017). Phenolic acids, including hydroxybenzoic and hydroxycinnamic acids, also exhibit distinct antioxidant activities (Alcalde et al. 2019). The hydroxyl groups integral to phenolic acids facilitate hydrogen atom donation, effectively neutralizing free radicals. This mechanism is particularly pronounced for the hydroxyl substituents on the aromatic ring. Moreover, phenolic acids engage in electron transfer, quenching the reactivity of free radicals and averting cellular damage (Flora 2009). Similar to flavonoids, phenolic acids enhance the expression of endogenous antioxidant enzymes, augmenting the cell's ability to combat oxidative stress (Zhang and Tsao 2016). In addition to flavonoids and phenolic acids, other polyphenolic compounds contribute to the antioxidant landscape. Stilbenes, typified by resveratrol, directly scavenge free radicals and attenuate oxidative stress (Tellone et al. 2015). They also exert anti‐inflammatory effects and modulate signaling pathways related to antioxidant enzyme expression (Hussein et al. 2016). Curcuminoids are abundant in turmeric, neutralize free radicals through hydrogen atom donation, and activate Nrf2 signaling, which promotes the transcription of antioxidant enzymes (Nimal et al. 2022; Sohn et al. 2021). Lignans found in seeds and grains exhibit antioxidant properties through radical scavenging and the strengthening of endogenous antioxidant defenses (Soengas Fernández et al. 2011). The antioxidant properties of flavonoids, flavones, phenolic acids, and other polyphenolic compounds are attributed to various intricate mechanisms. These compounds proficiently combat oxidative stress through direct radical scavenging, signaling modulation, the induction of endogenous antioxidants, and additional protective actions. Their multifaceted antioxidant attributes contribute to their well‐established roles in promoting health and preventing chronic diseases associated with oxidative damage.

### 3.1 Mechanisms of Antioxidant Action

**FIGURE 2:**
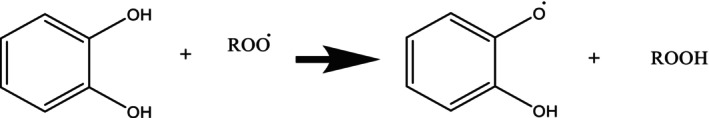
Catechol as an antioxidant and its mechanism of scavenging free radicals.

The antioxidant mechanisms of polyphenols include a multifaceted array of actions that collectively contribute to their potent ability to counteract oxidative stress (Lv et al. 2021). These mechanisms arise from the intricate structural features of the polyphenolic compounds and their interactions with biological systems. The generalized reaction that illustrates the scavenging of free radicals by antioxidants, such as polyphenols, is shown in Figure 2.

AH+R•→A•+H−R

**FIGURE 3:**
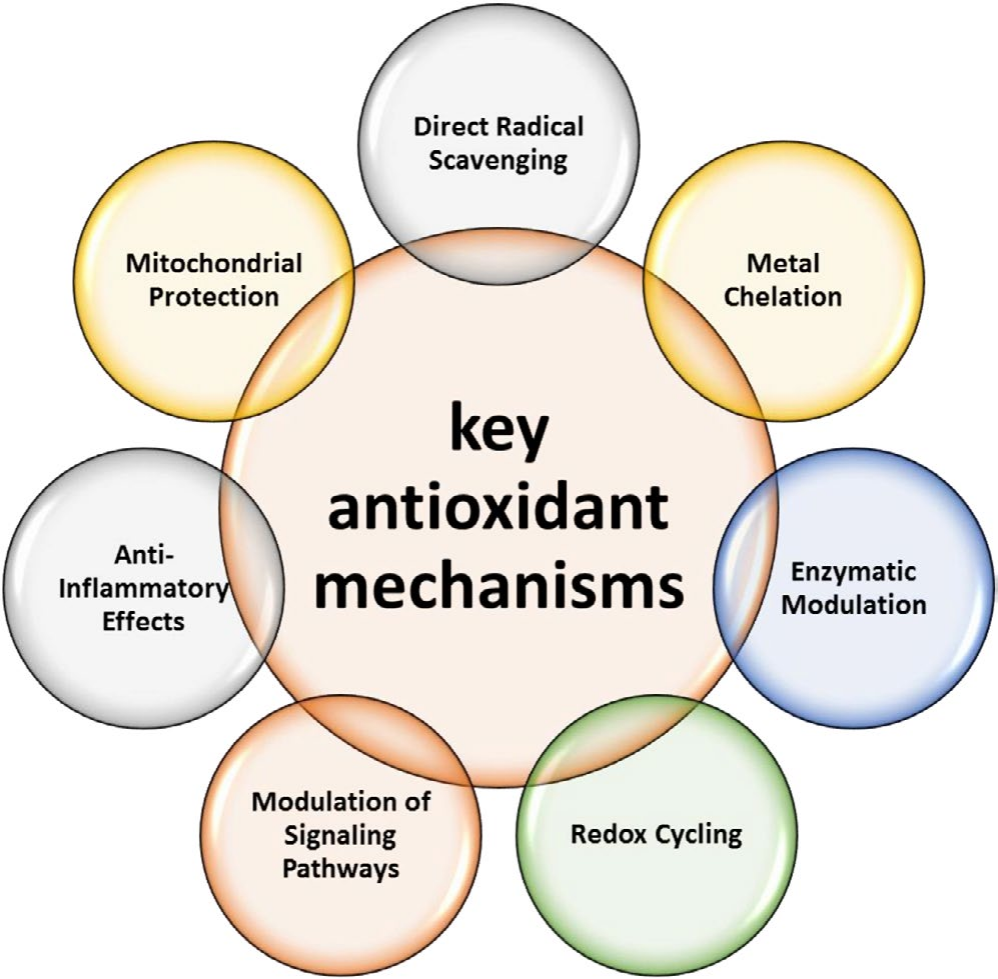
List of key antioxidant mechanisms of polyphenolic compounds.

In this reaction, an antioxidant (AH), polyphenol, or another type of antioxidant acts as the scavenger. Free radicals (R•) are reactive species that damage cells by receiving electrons from other molecules. The antioxidant radical (A•) donates an electron or hydrogen atom to the free radical, neutralizing its reactivity and leading to a stable molecule (H‐R) after the antioxidant scavenges the free radical. This stable molecule is no longer reactive and does not contribute to further oxidative damage. This reaction demonstrates how antioxidants, including polyphenols, act as electron or hydrogen atom donors for free radicals, preventing their harmful effects on cellular components and halting the chain reactions of oxidative stress. The fundamental antioxidant mechanisms are shown in Figure 3.

#### 3.1.1 Direct Radical Scavenging

Polyphenols can neutralize free radicals by acting as electron or hydrogen atom donors, thereby interrupting the chain reactions of oxidative damage. Hydroxyl groups and aromatic systems play pivotal roles in this process. Polyphenols, which donate electrons or hydrogen atoms, can inhibit the harmful effects of ROS and reactive nitrogen species (RNS) on cellular components, such as lipids, proteins, and DNA (Amić and Mastiľák Cagardová 2022; Di Meo et al. 2013).

#### 3.1.2 Metal Chelation

Certain polyphenols can chelate transition metal ions such as iron and copper (Ragan et al. 1979). These metals are responsible for the production of reactive oxygen species (ROS) through the Fenton and Haber‐Weiss reactions. By binding to these metals, polyphenols hinder their participation in ROS generation, subsequently reducing oxidative stress (Perron and Brumaghim 2009).

#### 3.1.3 Enzymatic Modulation

Polyphenols can affect the function of essential antioxidant enzymes such as SOD, catalase, and glutathione peroxidase. By upregulating these enzymes, polyphenols enhance cellular defense mechanisms against oxidative damage (Alvarez‐Suarez et al. 2011; Yan et al. 2020).

#### 3.1.4 Redox Cycling

Certain polyphenols possess the capacity to undergo redox cycling, where they can alternate between the oxidized and reduced states. This cycling allows them to intercept and neutralize ROS directly, thereby safeguarding cellular components from oxidative damage (Kagan and Tyurina 1998).

#### 3.1.5 Modulation of Signaling Pathways

Polyphenols can influence cellular signaling pathways associated with oxidative stress responses. They can activate the antioxidant response element (ARE) pathways, triggering the transcription of genes encoding antioxidant enzymes and other protective molecules (Costa et al. 2012).

#### 3.1.6 Anti‐Inflammatory Effects

Polyphenols exhibit anti‐inflammatory properties by inhibiting the activity of pro‐inflammatory enzymes and cytokines. By dampening chronic inflammation, they indirectly reduce the oxidative stress burden (Yahfoufi et al. 2018).

#### 3.1.7 Mitochondrial Protection

Polyphenols can safeguard mitochondria, the major cellular energy producers, and are the primary sites of ROS generation. Polyphenols significantly enhance mitochondrial function and mitigate oxidative damage within these organelles, thereby promoting cellular health (Camilleri et al. 2013). The diverse and intricate antioxidant mechanisms of polyphenols stem from their chemical structures, allowing them to dynamically interact with the oxidative challenges presented within biological systems. Polyphenols have been found to counteract oxidative stress, support overall health, and reduce the risk of chronic diseases associated with oxidative damage.

### 3.2 Critical Considerations in Antioxidant Evaluation

Most in vitro assays, such as DPPH, ABTS, and FRAP, measure the direct radical scavenging or reducing power in simplified systems, which may not accurately reflect the complexity of living organisms. On the other hand, the cell‐based assays provide more physiologically relevant insights but are strongly influenced by cellular uptake, metabolism, and bioavailability. In vivo assays, such as monitoring oxidative biomarkers or antioxidant enzyme activity in animal or human studies, offer the highest relevance, but are expensive and costly. Therefore, no single assay can comprehensively describe antioxidant potential, and a combined multi‐assay and in vivo approach is essential for a realistic understanding of biological significance (Amorati and Valgimigli 2015; Büyüktunce et al. 2014). Another critical aspect is the dose‐dependent dual role of the polyphenols. While low to moderate concentrations generally provide antioxidant benefits, excessive levels can exert pro‐oxidant effects. However, high doses of antioxidants and long‐term consumption may result in pro‐oxidant effects, leading to cell damage resulting in adverse effects instead of beneficial effects (Stoeva et al. 2025).

### 3.3 Relationship Between Polyphenolic Structure and Antioxidant Activity

The antioxidant efficacy of polyphenols is influenced by their chemical structures, including hydroxyl groups, conjugated double bonds, and aromatic rings, which contribute to electron and hydrogen atom donation. These structural features facilitate the interruption of the chain reactions initiated by ROS and RNS. For instance, multiple hydroxyl groups, such as those seen in flavonoids, such as quercetin and catechins, effectively enhance their capacity to neutralize free radicals. Additionally, the chelating ability of certain polyphenols for metal ions like iron and copper further augments their antioxidant potential (Rice‐Evans et al. 1996; Xue et al. 2014).

## 4 Methods for Isolating Polyphenolic Compounds from Plants

Extraction techniques are the foundational steps in isolating polyphenolic compounds from plant sources, unlocking their potential for various applications. These techniques are essential for liberating polyphenols from a complex matrix of plant materials and preparing them for further purification and characterization. This section delves into three key extraction methods: solvent extraction, supercritical fluid extraction (SFE), and solid‐phase extraction (SPE) (Alara et al. 2021).

### 4.1 Extraction Techniques

#### 4.1.1 Solvent Extraction

Solvent extraction is a cornerstone method because of its adaptability, efficiency, and widespread application. This technique involves immersing the plant material in a solvent with an affinity for polyphenols, thereby enabling the dissolution of these valuable compounds. Commonly used solvents include ethanol, methanol, and water, which are selected based on the polarity and properties of the target polyphenols (Rajbhar et al. 2015). Various methods can be used for solvent extraction. Maceration involves soaking the plant material in the chosen solvent for a specified period, gradually allowing the polyphenols to diffuse into the solvent (Masjedi et al. 2022). Alternatively, more intricate approaches, such as reflux or Soxhlet extraction, have been employed to achieve higher efficiency (Arias et al. 2009). During reflux, the solvent and plant material mixture was heated, causing the solvent to vaporize and condense back into the container, promoting the extraction process. In contrast, Soxhlet extraction employs a continuous cycle of solvent vaporization, condensation, and re‐percolation through the plant material, enhancing extraction efficiency (Arias et al. 2009). Solvent extraction is particularly effective in liberating polyphenols from diverse plant matrices because their solubility varies depending on their polarity, molecular weight, and functional groups. The outcome of solvent extraction is a crude extract enriched with a range of polyphenolic compounds, which serve as the initial material for subsequent purification and characterization steps (Alara et al. 2021).

#### 4.1.2 Supercritical Fluid Extraction (SFE)

Supercritical fluid extraction (SFE) is a novel technique that utilizes the unique properties of supercritical fluids, which exhibit characteristics of gases and liquids under specific temperature and pressure conditions (Sairam et al. 2012). Carbon dioxide (CO_2_) is a commonly employed supercritical fluid owing to its safety, nontoxicity, and tunable solvating power. In SFE, CO_2_ is pressurized to its supercritical state, which enables it to act as a solvent and carrier gas. Supercritical CO_2_ was then used to selectively extract polyphenols from the plant matrices. Upon depressurization, the supercritical CO_2_ returns to its gaseous state, leaving behind the extracted polyphenols (Sapkale et al. 2010). This method offers advantages, such as minimal thermal degradation due to low temperatures, making it suitable for heat‐sensitive compounds. SFE is particularly advantageous for the extraction of delicate and labile polyphenols because it avoids exposure to high temperatures and reduces the risk of residual solvent contamination. Its selectivity can be fine‐tuned by adjusting the pressure and temperature, making it a versatile tool for polyphenol extraction (Alara et al. 2021).

#### 4.1.3 Solid‐Phase Extraction

Solid‐phase extraction (SPE) is a precise and selective technique designed to concentrate and purify polyphenols from complex mixtures (Ötles and Kartal 2016). This method leverages the principle of adsorption and involves passing a liquid sample containing polyphenols through a solid phase, often a cartridge, that contains a sorbent material. The sorbent selectively retains the target polyphenols while allowing unwanted compounds to pass through. The retained polyphenols were eluted from the solid phase using a suitable solvent, resulting in a concentrated and enriched sample of polyphenols (Rodrıguez et al. 2000). Solid‐phase extraction is particularly beneficial for isolating specific polyphenols from matrices containing numerous interfering compounds. Its selectivity, reproducibility, and precision make it an essential tool for the preparation of purified polyphenolic samples for subsequent analyses and applications (Alara et al. 2021). An overview of the comparison between each extraction method is presented in Table 2. The selection of extraction techniques depends on the desired polyphenol subclass, cost, and environmental constraints. Green chemistry approaches, including solvent recovery and CO_2_‐based extraction, are becoming increasingly important for sustainable production.

**TABLE 2:** Comparative analysis of the polyphenol extraction techniques.

Method	Yield efficiency	Scalability	Cost	Environmental impact	Notes
Solvent extraction	High	High	Low	Solvent residues, less eco‐friendly	Widely used, versatile
Supercritical fluid (SFE)	Moderate–High	Medium	High	Green, minimal residues	Preserves heat‐sensitive compounds
Solid phase extraction	Very selective	Low–Medium	Moderate	Eco‐friendly, low waste	Best for purification, not bulk yield

### 4.2 Characterizations and Purification Techniques

Numerous studies have been published on the separation, purification, and identification of phenolic compounds in plants, which have led to numerous improvements in this field. These protocols and procedures can identify and isolate compounds with high purity and precision. Phenolic compounds in plants have been purified and identified using various protocols and spectroscopic techniques, such as UV–visible spectroscopy, NMR, and HPLC, because of their unique structures and properties (Ajila et al. 2011; Jahromi 2019). The methods used for the separation, purification, and identification of these compounds are described below.

#### 4.2.1 UV–Visible Spectroscopy

UV–visible spectroscopy is a spectroscopic technique used to measure absorption within the UV–visible spectral range, which corresponds to the electronic transitions within molecules. This approach is particularly pertinent because the aromatic structure inherent to phenolic compounds endows them with potent chromophores in the ultraviolet (UV) range. Flavones, phenolic acids, and total anthocyanins exhibit absorption spectra at 320, 360, and 520 nm, respectively, highlighting their unique properties. The efficacy of this method lies in its brevity, affordability, and reproducibility, rendering it a favorable choice in analytical practice. Nonetheless, the efficacy of UV–visible spectroscopy can be used in scenarios characterized by partially overlapping peaks, which impede accurate identification. To overcome this challenge, a strategic tandem approach has been proposed. By combining UV–visible spectroscopy with complementary techniques such as mass spectrometry or HPLC, researchers can unravel complex profiles, ensuring enhanced accuracy and comprehensive insight into the composition of polyphenolic compounds. This holistic approach embraces the strengths of various analytical tools, empowering a deeper understanding of the polyphenol content and composition, thereby enriching the scope of scientific exploration and industrial applications (Ajila et al. 2011; Jahromi 2019).

#### 4.2.2 Infrared Spectroscopy

IR radiation typically has wavenumbers between 4000 and 600 cm^−1^, corresponding to wavelengths of 2.5–17 μm. IR, which is divided into near‐infrared, mid‐infrared, and far‐infrared regions, effectively identifies molecules by analyzing vibrational shifts within them, revealing their functional groups. The vibrational frequencies associated with different functional groups, including single, double, triple, carboxyl, hydroxyl, and amino bonds, emerged as distinctive signatures within the spectra. However, it is important to note that infrared spectroscopy does not directly depict the structural arrangement of chemical compounds. Rather, its focus lies in unraveling the essence of the functional groups embedded within the molecular framework (Jahromi 2019). A study of the mid‐infrared spectra of 36 standard phenolic compounds provided a compelling example of this potential. The MIR spectral analysis demonstrated a remarkable ability to differentiate between the flavonoid and phenolic acid families. In a noteworthy conclusion, Abbas and colleagues underscored the potential for enhanced precision by broadening the scope of the investigation to encompass a more expansive array of phenolic compound samples, thus deepening the discriminative power of this analytical approach. IR spectroscopy, incapable of rendering structural blueprints, profoundly influences the functional tapestry of molecules. Vibrational nuances and spectral patterns offer a gateway for understanding the inherent nature of functional groups within chemical structures, potentially revolutionizing our understanding of complex molecular landscapes (Abbas et al. 2017).

#### 4.2.3 Nuclear Magnetic Resonance

NMR is a technique that relies on the detection of electromagnetic radiation within the radio‐wave spectrum. Unlike FTIR and UV–visible spectroscopic techniques, which rely on electron absorption, NMR centers on the magnetic characteristics of the atomic nuclei. Although ^1^H NMR and ^13^C NMR are conventionally used to examine the chemical structures of materials, this approach can be extended to nuclei with spin properties. In addition to discerning chemical compounds, this spectroscopic approach also provides precise insights into the molecular structure, dynamics, reaction states, and chemical surroundings. Numerous publications have underscored the utility of ^1^H NMR spectroscopy in effectively identifying and scrutinizing polyphenolic compounds in plant matter. Moreover, this rapid, quantitative, and non‐destructive method offers substantial benefits in this context (Jahromi 2019). Christophoridou and Dais (2009) has conducted a study using high‐resolution ^1^H NMR to detect and quantify the phenolic compounds in olive oil. This study's methodology for identifying phenolic compounds relies on exploiting NMR chemical shifts and leveraging an extensive library of model compounds meticulously assigned using two‐dimensional (2D) NMR spectroscopy techniques. Additionally, the application of 2D NMR has been extended to examine phenolic extracts in order to uncover novel phenolic moieties. The ^1^H NMR method has proven useful in detecting and characterizing various phenolic entities, from uncomplicated ones such as p‐coumaric acid, to more complex constituents such as flavonols, lignans, and isomers of oleuropein and ligstroside. Notably, the quantification of total hydroxytyrosol and total tyrosol, encompassing free and esterified forms, was achieved, providing a comprehensive representation of their presence. For accurate quantification, anhydrous 1,3,5‐triazine was used as an internal standard within the polar fraction (Christophoridou and Dais 2009).

#### 4.2.4 High‐Performance Liquid Chromatography

This chromatographic method is highly effective for partitioning, characterizing, and measuring polyphenolic compounds. In this process, the use of a pressurized mobile phase separates the constituent mixtures into a stationary phase. HPLC is the preferred method for separating and quantifying phenolic compounds using gas chromatography (GC). Along this line, diverse factors play a role in HPLC efficacy, including aspects such as sample refinement, mobile phase characteristics, column types, and detector configurations. HPLC is widely used to purify and quantify phenolic compounds, typically using a normal‐phase C18 or reversed‐phase column with solvents of varying polarities. The pH of the mobile phase should be stabilized within the 2–4 range to prevent ionization of phenolic compounds. An aqueous mobile phase with acidification is crucial for precise purification and quantification of phenolic compounds (Jahromi 2019). Recently, a study validated an approach for quantitatively determining the concentration of two compounds that exhibit similar retention times, and thus manifest overlapping peaks within a mixed solution. These researchers examined two phenolic compounds: caffeic acid and vanillic acid, and ferulic and *p*‐coumaric acids. This method capitalizes on the distinct absorbance behavior of the two phenolic compounds at different wavelengths within the eluent. Remarkably, this method enabled quantitative determination of these compounds, even in instances where they were not fully resolved within the HPLC column. The method described here holds promise for deciphering the outcomes of HPLC analyses of food products containing a wide spectrum of phenolic compounds and flavonoids. Its applicability could significantly enhance the interpretation of such complex analyses (Mizzi et al. 2020).

## 5 Sources of Polyphenolic Compounds with Antioxidant Potential

Polyphenolic compounds, which have potent antioxidant properties, are abundant in a diverse array of plant‐based sources and contribute remarkable health benefits associated with natural diets. These compounds serve as defenders against oxidative stress and cellular damage induced by ROS and free radicals (Li et al. 2014).

### 5.1 Fruits and Berries

Phenolic substances are abundant secondary metabolites in fruits, with flavonoids and phenolic acids accounting for most of these metabolites. The growing interest in these chemicals is primarily due to their antioxidant properties and their potential to prevent certain illnesses through ingestion. The health benefits of phytochemicals are significantly affected by their frequent consumption and their bioavailability. Studies have indicated that regular fruit consumption is beneficial, particularly for reducing illnesses associated with oxidative stress (Haminiuk et al. 2012). Fruits and berries are primary reservoirs of polyphenols and contain a spectrum of antioxidant‐rich compounds. Blueberries, strawberries, raspberries, blackberries, and cherries, enriched with anthocyanins, flavonols, and other polyphenols, not only impart vibrant types, but also deliver substantial antioxidant benefits, reducing oxidative stress (Paśko et al. 2021).

### 5.2 Citrus Fruits

The world is a hub for citrus fruits, which are consumed in various forms such as fresh, processed, and cooked juices and pies. The world's economic relevance is evident, as the global output of CF commodities is expected to reach approximately 98 million tons by 2021. CF is a rich source of phenolic compounds (PC) owing to its abundance and diversity. Recent research suggests that consuming citrus fruits may help prevent chronic diseases and cancer, despite the ancient association between PC and oxidative stress diseases (Sanches et al. 2024). Citrus fruits, including oranges, grapefruits, lemons, and limes, are rich in flavonoids like hesperidin, naringenin, and quercetin, which have potent antioxidant properties (Zou et al. 2016).

### 5.3 Apples

Apples, a dietary staple, contain an assortment of polyphenols, such as flavonols (such as quercetin), catechins, and procyanidins. These compounds contribute to their antioxidant potential, and have been linked to various health‐enhancing effects (Biedrzycka and Amarowicz 2008). Apples have garnered increasing scientific attention owing to several studies demonstrating their advantageous impact on human health. Apples contain antioxidant components that reduce the risk of degenerative and cardiovascular diseases, which are influenced by oxidative stress, especially free radicals and reactive oxygen species (Hyson 2011). Owing to heightened ROS exposure, antioxidants have become the subject of extensive study. Polyphenols, which are the predominant antioxidants found in apples, serve as the most plentiful antioxidants in the human diet (Scalbert et al. 2002; Tsao et al. 2005). Apples, which are widely used and available year‐round, are a significant source of dietary polyphenolic compounds in the western diet owing to their widespread use (Wolfe et al. 2003).

### 5.4 Grapes and Wine

Grapes, particularly the red variety, are a significant source of polyphenols, most notably resveratrol (resveratrol). This compound and other polyphenols contribute to the health‐promoting properties of grapes and their derivatives, which are associated with cardiovascular benefits (Latruffe and Rifler 2013). Non‐volatile phenolic compounds and derivatives are natural components of grapes and related products, particularly wine. Phenolic acids, flavonoids, tannins, stilbenes, coumarins, lignans, and phenylethanol analogs are members of a diverse family of chemical substances. Phenolic compounds significantly influence the sensory characteristics of grapes and wine, including fragrance, color, taste, bitterness, and astringency, owing to their organoleptic properties (Linskens and Jackson 1988; Macheix and Fleuriet 1993). The non‐volatile phenolic composition of wine is influenced by factors such as grape variety, maturity, vineyard environment, winemaking technology, fermentation, and aging conditions (Fang et al. 2008). Pre‐fermentative practices, such as the addition of SO2 and ascorbic acid, maceration, alcoholic fermentation, yeast strain inoculation, malolactic fermentation, precipitation, oxidation, adsorption, β‐glucosidase activity, and clarification with fining agents, influence phenolic compound levels (Balík et al. 2008; Kennedy 2008).

### 5.5 Teas

Teas, particularly green and black teas, are rich in polyphenols including EGCG, which are antioxidants known for their ability to combat oxidative stress and promote overall health (Dreosti 1996). Phenolic chemicals, such as theaflavins (TF), thearubigins (TR), and brownies (TB), are the primary indicators of tea product quality. A study of 56 leaf teas and teabags from Queensland supermarkets revealed varying levels of TF, with low concentrations of black tea, possibly due to excessive fermentation or prolonged storage. The solubilities of TR and TB in teabags vary during storage, indicating potential instability and oxidation. The pricing of green tea has shown substantial variability in the total polyphenol content, ranging from 14% to 34% (Yao et al. 2006).

### 5.6 Cocoa and Dark Chocolate

Cocoa and its delightful derivative, dark chocolate, contain many flavonoids, especially flavanols, such as epicatechin. These compounds exhibit potent antioxidant effects and are attributed to potential cardiovascular protection associated with moderate consumption of dark chocolate (Vinson et al. 1999). Polyphenols, which are important components of cocoa and dark chocolates, have been linked to positive health outcomes when consumed as food. Cocoa and dark chocolate polyphenols have anti‐inflammatory and antioxidant properties, activating key signaling pathways, causing vasodilation and cardioprotective benefits through nitric oxide production (Magrone et al. 2017).

### 5.7 Spices

The aromatic world of spices is not only related to flavor, but also to potent polyphenols. Turmeric, which is renowned as curcumin, has anti‐inflammatory and antioxidant properties. Cinnamon and cloves, rich in polyphenols, contribute their beneficial attributes, including antioxidant and anti‐inflammatory effects (Yashin et al. 2017). This study used ultra‐performance liquid chromatography and a photodiode array detector to analyze the antioxidant capacity, total phenolic content, and major phenolic compounds of 19 commonly consumed spices in China. Galangal, a spice with high antioxidant capacity, is primarily composed of galangin, whereas the Rutaceae and Lauraceae species also exhibit high antioxidant capacity and phenolics. The study reveals that the predominant phenolic compounds in spice extracts are chlorogenic acid and rutin, offering potential insights into human health and the development of new natural antioxidants (Lu et al. 2011).

### 5.8 Nuts

The most commonly consumed nuts worldwide are almonds, Brazil nuts, cashews, hazelnuts, macadamia nuts, pecans, pine nuts, pistachios, and walnuts, which can be eaten unprocessed or processed. The health benefits of natural nuts are attributed to their phenolic profiles, total phenolic levels, and antioxidant activity, particularly in the kernels and skin. Roasting removes the skin, alters phenolic chemicals and antioxidant activity, and is particularly effective in preserving food. Nut skins, which are rich in phenolic compounds, have been re‐examined in the food, pharmaceutical, and cosmetic industries (Taş and Gökmen 2017). Nuts, such as walnuts, pecans, and almonds, contain notable polyphenols, such as ellagic acid and resveratrol. These compounds contribute to the nuts' reputation as healthful snacks, offering antioxidant and potential health‐promoting effects (Vinson and Cai 2012).

### 5.9 Whole Grains

Among the main food sources for humans are cereal grains, the production of which has expanded in recent years to meet the global demand. Wheat is the most widely consumed whole grain, constituting a large portion of the human diet. Because of their constituent portions, bran and germ, which contain special health‐promoting bioactive ingredients, whole grains have excellent nutritional and bioactive qualities. The World Health Organization's 2012–2016 report and evidence of health benefits from human intervention studies encourage the consumption of whole grains and whole‐grain meals in the diet. Multiple epidemiological studies have highlighted the inverse relationship between eating whole grains and lower incidence of metabolic syndrome and chronic diseases (Călinoiu and Vodnar 2018). Beyond their fiber and nutrient contents, whole grains such as oats, barley, and quinoa contain polyphenols such as ferulic acid and lignans. These compounds boost the antioxidant potential of entire grains, contributing to their role in well‐rounded diets (Wu et al. 2017).

### 5.10 Legumes

Legumes are rich in bioactive phenolic compounds that are crucial in various physiological and metabolic processes. Beans, lentils, and peas pack antioxidant punches through their flavonoid, tannin, and other polyphenolic constituents. These compounds contribute to their anti‐inflammatory effects and overall health benefits (Salunkhe et al. 1983). This study examined the phenolic content of 29 mature European grain legume seeds, including chickpeas, field peas, faba beans, common vetch, and lupins. This study revealed that phenolic acids were more prevalent in chickpeas, field peas, and common vetch than flavonoids, whereas the opposite was observed in lupin seeds. Yellow lupins and narrow‐leaf lupins had the highest total phenolic levels, whereas Kabuli chickpeas had the lowest. Flavones and total phenolic compounds accounted for 51% of the data variability (Magalhaes et al. 2017).

### 5.11 Herbs

Herbs, beyond their culinary allure, contain a range of polyphenols. Rosemary, thyme, oregano, and sage contain polyphenols such as rosmarinic acid and flavonoids, which have antioxidant and antimicrobial properties (Okuda 1999). This study analyzed the antioxidant capacities and phenolic contents of 27 culinary and 12 medicinal herbs, and found that medicinal herbs had higher ORAC and phenolic contents. Culinary herbs such as *Catharanthus roseus* , *Thymus vulgaris* , *Hypericum perforatum* , and *Artemisia annua* exhibited higher ORAC values and total phenolic contents. A linear relationship was found between ORAC values and total phenolic content in selected herbs using HPLC and diode‐array detection to identify and quantify phenolic compounds. Rosmarinic acid is the predominant phenolic compound in *Salvia officinalis* , *Thymus vulgaris* , Origanum majoricum, and *P. longiflora* , whereas quercetin‐3‐O‐rhamnosyl‐(1 → 2)‐rhamnosyl‐(1 → 6)‐glucoside and kaempferol‐3‐O‐rhamnosyl‐(1 → 2)‐rhamnosyl‐(1 → 6)‐glucoside are predominant in *Ginkgo biloba* leaves (Zheng and Wang 2001) A study examined the antioxidant capacities and phenolic contents of 32 spice extracts from 21 Polish botanical families, estimating the total antioxidant capacity using ABTS radical dot+, DPPH radical dot, and ferric reducing/antioxidant power. Phenolics were measured using the Folin–Ciocalteu assay, which revealed major phenolic acids such as caffeic, p‐coumaric, ferulic, and neochlorogenic acids, and predominant flavonoids such as quercetin, luteolin, apigenin, kaempferol, and isorhamnetin (Wojdyło et al. 2007).

### 5.12 Vegetables

The quality of food obtained from plants is significantly influenced by phenolic secondary metabolites, which affect aspects such as flavor, appearance, and health‐promoting qualities.

Numerous factors that affect phenolic stability, production, and degradation also affect the food content. The main enzyme, phenylalanine ammonia‐lyase (PAL), is particularly important for biosynthesis because it can be triggered by many stressors (environmental conditions). The primary enzymes responsible for the quality loss caused by phenolic degradation are polyphenol oxidase (PPO) and peroxidase (POD) (Tomás‐Barberán and Espín 2001). Leafy greens, such as spinach and kale, cruciferous vegetables, such as broccoli, and vibrant red cabbage are troves of polyphenols, such as quercetin and kaempferol. These compounds are essential for the antioxidant properties and potential health benefits of these vegetables (Panja 2018). A study analyzed the phenolic composition of various vegetables consumed by African Americans in the southeast US using HPLC–MS, including collard greens, mustard greens, kale, okra, sweet potato greens, and eggplant. Five compounds were identified from the 29 peaks: caffeic acid, ferulic acid, quercetin, kaempferol, and isorhamnetin, whereas no gallic acid, p‐coumaric acid, myricetin, luteolin, apigenin, hesperetin, naringenin, or flavonols were detected. Isorhamnetin, quercetin, and kaempferol are the major flavonoids found in kale, mustard green, purslane, collard green, mustard green, kale, okra, sweet potato green, purple‐hull peas, and purslane. The study indicates that indigenous vegetables are effective sources of phenolic compounds, which may be beneficial for preventing cardiovascular and chronic diseases (Huang et al. 2007).

### 5.13 Olive Oil

Extra virgin olive oil, a key component of the Mediterranean diet, contains unique polyphenols such as hydroxytyrosol and oleuropein. These compounds underscore the well‐documented antioxidant and anti‐inflammatory effects of the oil, making it an essential culinary choice (Gorzynik‐Debicka et al. 2018). The Mediterranean diet has beneficial biological effects and is rich in fruits, vegetables, grains, dairy products, fish, wine, and olive oil. Epidemiological studies have revealed a decrease in cardiovascular illnesses, atherosclerosis, and various types of cancer in the Mediterranean region. The health benefits of the Mediterranean diet are attributed to the high unsaturated to saturated fatty acid ratio of olive oil and its antioxidant properties, which are primarily derived from its phenolic components. The primary phenolic substances that give extra‐virgin olive oil its strong, bitter flavor are hydroxytyrosol and oleuropein, which also exhibit potent antioxidant properties both in vivo and in vitro (Tripoli et al. 2005). The Mediterranean diet, including olive oil, is being studied for cancer prevention owing to its health benefits, including its phenolic antioxidants that inhibit reactive oxygen species attack. Reactive oxygen species, linked to fat‐related neoplasms such as breast and colorectal cancer, may be triggered by a high intake of ω‐6 polyunsaturated fatty acids. Researchers have developed high‐performance liquid chromatography (HPLC) methods to quantify the reactive oxygen species generated by the fecal matrix. Studying the correlation between reactive oxygen species and dietary antioxidants holds the potential for understanding colorectal carcinogenesis mechanisms and devising future chemopreventive strategies (Owen et al. 2000).

### 5.14 Onions and Garlic

Garlic and onion are widely used dietary components that have been shown to have potential health advantages, such as their ability to combat cardiovascular disease, diabetes, blood clotting, and hyperhomocysteinemia. Furthermore, they have antibacterial, antioxidant, anticarcinogenic, antimutagenic, antiasthmatic, immunomodulatory, and prebiotic properties (Corzo‐Martínez et al. 2007). Onions and garlic, which are cherished for their culinary versatility, contain organosulfur compounds and flavonoids. These compounds showcase antioxidant and potential health‐enhancing effects, further adding to the appeal of these culinary essentials (Kavalcová et al. 2014). This study assessed the antioxidant activity and total polyphenol content of onion and garlic samples from a village in Erbil City. The study revealed that red onions exhibited the highest total polyphenol content and antioxidant activity, whereas white onions had the lowest. The study found that red onions had the highest total polyphenolic compound content, followed by garlic, yellow, pink, and white onions. Garlic and onion are regarded as health‐promoting agents because of their high phenolic content and antioxidant activity (Dalaram 2016).

### 5.15 Red Beans

Red beans, encompassing kidney beans and red lentils, harbor anthocyanins and a range of polyphenols. These compounds have antioxidant properties and nutritional value (Yang et al. 2018). Red beans contain bioactive polyphenols, which can potentially modify the toxic effects of mycotoxins. According to a Q‐Orbitrap study, epicatechin and delphinidin are the most frequently detected polyphenols in red bean extracts. The extract was tested for T‐2 toxin‐induced cytotoxicity in HepG2 cells, which showed that T‐2 affected cell viability and increased ROS production. Red bean extract significantly reduced ROS production, suggesting its potential to mitigate oxidative stress in HepG2 cells (Martínez‐Alonso et al. 2022). The main dietary sources, predominant compounds, and key functional outcomes are summarized in Table 3.

**TABLE 3:** Major dietary sources of polyphenols, predominant classes, and health implications.

Source	Major polyphenols	Typical effects on health	Processing/bioavailability notes
Fruits & berries	Anthocyanins, flavonols, phenolic acids	Cardioprotection, reduced oxidative stress	Anthocyanins sensitive to pH, heat, storage
Tea (green/black)	Catechins (EGCG), theaflavins	Antioxidant, anti‐inflammatory, metabolic regulation	Fermentation alters catechin profile
Grapes & wine	Resveratrol, flavonols, tannins	Cardiovascular protection, anti‐aging	Alcohol matrix improves resveratrol absorption
Cocoa & chocolate	Flavanols (epicatechin, catechin)	Vascular function, cognitive health	Roasting decreases catechins
Olive oil	Hydroxytyrosol, oleuropein	Anti‐inflammatory, metabolic benefits	Stability depends on storage
Legumes	Tannins, phenolic acids	Anti‐inflammatory, gut health support	Cooking reduces free phenolics
Whole grains	Ferulic acid, lignans	Antioxidant, anti‐diabetic	Bound forms require gut microbiota release
Nuts	Ellagic acid, resveratrol, flavonoids	Cardioprotective, anti‐inflammatory	Skin richest in phenolics; roasting alters content
Spices & herbs	Curcumin, rosmarinic acid, cinnamic acid derivatives	Anti‐inflammatory, antimicrobial	Bioactive retention varies with drying, extraction
Vegetables	Quercetin, kaempferol, isorhamnetin	Antioxidant, anti‐cancer potential	Polyphenol oxidase reduces content post‐harvest

The availability and stability of dietary polyphenols depend on the food matrix and processing. For example, anthocyanins are highly sensitive to heat and pH, whereas bound phenolics in cereals are released only after microbial metabolism in the gut. Practices such as fermentation, roasting, or boiling can significantly alter polyphenol profiles, thereby modifying their health effects. It should also be noted that variability in the polyphenol content has been reported across studies. Some sources provide quantitative ranges, whereas others only list the presence or absence. This inconsistency complicates the direct comparisons between food groups. Furthermore, the effects of processing, storage, and preparation are often underreported despite their substantial impact on polyphenol retention. Standardization of reporting methods and systematic evaluation of processing effects would significantly enhance cross‐study comparisons and dietary recommendations.

## 6 Conclusion and Future Perspectives

Polyphenolic compounds represent a diverse and powerful class of natural antioxidants with wide‐ranging implications in nutrition, medicine, and industry. This comprehensive review provides a thorough and insightful exploration of the antioxidant potentials of polyphenolic compounds. By highlighting the diverse sources of polyphenols in the human diet and their mechanisms of action, we underscored their importance in promoting overall health and combating oxidative stress. Examination of sources, including fruits, vegetables, beverages, and spices, underscores their wide‐ranging prevalence in the human diet and plays a pivotal role in promoting overall health. Additionally, state‐of‐the‐art characterization techniques have demonstrated progress in unraveling the complexities of polyphenols, which are crucial for advancing our understanding of their antioxidant properties. A critical analysis of the current limitations and challenges provides a realistic perspective in the field, guiding future research endeavors to overcome obstacles and achieve further advancements. This review contributes significantly to the knowledge of polyphenolic antioxidants, emphasizing their multifaceted health benefits and the need for continued exploration in this captivating study area. Moreover, the versatility of polyphenols opens up opportunities for their integration into a wide array of industries, from pharmaceuticals to functional foods. This interdisciplinary approach fosters innovation and promotes collaboration among experts from different fields, thereby enriching the exploration of polyphenols' capabilities. Developing personalized interventions tailored to individual needs has become increasingly feasible as we continue to uncover the intricate relationships between polyphenols and human health. This could revolutionize preventive healthcare and therapeutic strategies, potentially leading to more effective treatments for various ailments. The potential of polyphenolic antioxidants to improve human health and well‐being is vast, and ongoing research and collaboration are crucial to unlocking their full potential. Despite advancements in our understanding of polyphenolic chemicals, several areas require further research. Future research should focus on improving the extraction and purification methods to enhance efficiency and minimize environmental impacts. Green chemistry advancements and sustainable practices may lead to eco‐friendly techniques for polyphenol extraction. To better understand the physiological effects of polyphenols, more thorough research on their absorption and metabolism in the human body is required. Advancements in polyphenolic compound identification could lead to more precise assessments of the antioxidant potential through the development of new analytical instruments and technologies. Finally, investigation of the synergistic effects of polyphenols and other bioactive substances may lead to new therapeutic and preventive interventions. Further research is required to fully utilize polyphenolic compounds to improve human health.

## Author Contributions

Z.A.: conceptualization, investigation, writing – original draft, writing – review, and editing. A.R.: supervision, conceptualization, investigation, writing – original draft, writing – review, and editing. M.S.M. and M.T.: writing – review, and editing. I.E.O., M.T., L.T., Z.E., B.K.K., and Z.A.: writing – review. All authors read and approved the submitted version.

## Funding

The authors have nothing to report.

## Ethics Statement

The authors have nothing to report.

## Consent

The authors have nothing to report.

## Conflicts of Interest

The authors declare no conflicts of interest.

## Data Availability

The authors have nothing to report.
